# What to expect from the price of healthy and unhealthy foods over time? The case from Brazil

**DOI:** 10.1017/S1368980019003586

**Published:** 2020-03

**Authors:** Emanuella Gomes Maia, Camila Mendes dos Passos, Renata Bertazzi Levy, Ana Paula Bortoletto Martins, Laís Amaral Mais, Rafael Moreira Claro

**Affiliations:** 1 Nursing Postgraduate Program, Federal University of Minas Gerais, Avenue Professor Alfredo Balena 190, Santa Efigênia, Belo Horizonte, MG 30130-100, Brazil; 2 Department of Health Sciences, State University of Santa Cruz, Ilhéus, BA, Brazil; 3 Department of Medicine and Nursing, Federal University of Viçosa, Viçosa, MG, Brazil; 4 Department of Preventive Medicine, University of São Paulo, São Paulo, SP, Brazil; 5 Brazilian Institute of Consumer Protection, São Paulo, SP, Brazil; 6 Department of Nutrition, Federal University of Minas Gerais, Belo Horizonte, MG, Brazil

**Keywords:** Food prices, Time trends, Ultra-processed foods, Chronic disease, Public health

## Abstract

**Objective:**

To measure change in price of food groups over time (1995–2030) in Brazil, considering the Brazilian Dietary Guidelines’ recommendations.

**Design:**

Data from the Household Budget Survey (2008–2009 HBS) and the National System of Consumer Price Indexes (NSCPI) were used to create a data set containing monthly prices for the foods and beverages most consumed in the country (*n* 102), from January 1995 to December 2017. Data on price of foods and beverages from 2008–2009 HBS (referring to January 2009) were used to calculate real price over time using the monthly variation in prices from NSCPI. All prices were deflated to December 2017. Foods and beverages were classified following the Brazilian Dietary Guidelines’ recommendations. The monthly price for each food group and subgroup was used to analyse changes in prices from 1995 to 2017 and to forecast prices up to 2030 using fractional polynomial models.

**Setting:**

Brazil.

**Participants:**

National estimates of foods and beverages purchased for Brazil.

**Results:**

In 1995, ultra-processed foods were the most expensive group (R$ 6·51/kg), followed by processed foods (R$ 6·44/kg), then unprocessed or minimally processed foods and culinary ingredients (R$ 3·45/kg). Since the early 2000s, the price of ultra-processed foods underwent successive reductions, becoming cheaper than processed foods and reducing the distance between it and the price of the other group. Forecasts indicate that unhealthy foods will become cheaper than healthy foods in 2026.

**Conclusions:**

Food prices in Brazil have changed unfavourably considering the Brazilian Dietary Guidelines’ recommendations. This may imply a decrease in the quality of the population’s diet.

Non-communicable diseases (NCD) are the main cause of death and disability in the world^(1)^. In 2016, NCD accounted for four out of every six deaths worldwide (71 %), mainly affecting low- and middle-income countries, with about 48 % of deaths occurring before the age of 70 years^(1)^. Due to growing burdens of NCD and rising rates of risk exposure, Brazil and 192 Member States of the UN adopted the Sustainable Development Goals as a guideline for the development of national policies and activities of international cooperation^(2)^. By 2030, these countries aim to have reduced premature mortality from NCD by one-third, through prevention and treatment^(2)^.

Healthy food consumption is a central factor in tackling NCD^(3,4)^. The dietary pattern associated with higher NCD risk is characterized by the high consumption of ultra-processed foods (such as soft drinks and salty snacks) in parallel with the insufficient consumption of unprocessed or minimally processed items (such as fruits, vegetables and beans). Ultra-processed foods have a negative impact on health not only due to their nutrient profile (high in sodium, free sugar and total fat; low in fibre, vitamins and minerals), but also a series of mechanisms developed to produce overconsumption^(5)^. Evidence indicates that these products are gradually becoming dominant in the global food system^(6,7)^. In Brazil, this growing amount of ultra-processed foods in the population’s diet has been observed in the last decades^(8)^.

In 2014, the Ministry of Health of Brazil published the second edition of the Brazilian Dietary Guidelines^(9)^. This was the first set of dietary guidelines to consider the influence of the industrial processing of foods on health, recommending the consumption of unprocessed or minimally processed foods instead of ultra-processed ones. The Brazilian Dietary Guidelines recognize several barriers for the adoption of a healthy diet, with special emphasis on food prices^(9)^. Unlike the situation observed in developed countries^(10,11)^, in Brazil a diet based on unprocessed or minimally processed foods such as grains (e.g. rice and beans) is still cheaper than one based on ultra-processed foods^(12)^. However, as the price of food groups changes over time with different intensities^(13,14)^, the long-term maintenance of this scenario is unclear. Evidence suggests that unhealthy foods and beverages are becoming increasingly more affordable than the healthy alternatives in developing countries^(15)^; however, no comprehensive study considering the framework established by the Brazilian Dietary Guidelines is available so far.

In this context, the present study aimed to measure change in price of food groups over time (1995–2030) in Brazil, considering the Brazilian Dietary Guidelines’ recommendations. The central hypothesis is that the price gap between healthy and unhealthy foods has decreased over time. Therefore, the study intended to improve knowledge regarding the barriers for the adoption of healthy diets in the country (and similar settings) and also to contribute to the improvement of public policies and regulatory measures directed towards the promotion of healthy eating.

## Methods

### Methods summary

Data from the most recent national Household Budget Survey (2008–2009 HBS) and from the National System of Consumer Price Indexes (NSCPI), both publicly available and collected by the Brazilian Institute of Geography and Statistics, were used to create a novel data set containing monthly prices (R$/kg) for the foods and beverages most consumed in the country (*n* 102) between January 1995 and December 2017. NSCPI does not provide actual price data, only monthly variation in prices. Thus 2008–2009 HBS unit prices were used to calculate prices from 1995 to 2017 using the monthly variation from NSCPI. The same basket of foods with 102 items was used uniformly over time to allow for comparison of these prices. All prices were deflated (according to the official national inflation index) to represent December 2017 values. The foods and beverages were classified following the Brazilian Dietary Guidelines’ recommendations (NOVA classification system) into four groups and seventeen subgroups. The mean monthly price (R$/kg) for each food group and subgroup was estimated considering the acquired amount (in kilograms) of these 102 foods and beverages according to 2008–2009 HBS. These values were used to analyse changes in price for the period from 1995 to 2017 and to forecast price values up to 2030 (using fractional polynomial models).

Analyses involving food prices per unit of energy (i.e. R$/1000 kcal; 1 kcal = 4·184 kJ) are available in the online supplementary material.

### Food prices and food consumption data

HBS have been periodically conducted (generally once per decade) in Brazil since the 1970s^(16)^. The 2008–2009 edition is the most recent one with available information up to the moment of conclusion of the present study (a new survey was conducted in 2017–2018 but data remain unavailable). The structure of consumption identified in these studies (based on families’ expenditures and income) serves as the framework for the consumer price indexes estimated in the country. The 2008–2009 HBS used a two-stage cluster sampling strategy, with the random selection of census tracts in the first stage and of households in the second stage. All 12 800 census tracts of the country (information obtained from the 2000 Demographic Census) were previously grouped to obtain household strata with high geographical and socio-economic homogeneity, constituting 550 household strata. Census tracts were then randomly selected from each stratum, proportionally considering the number of households in the stratum. Households from each tract were selected by simple random sampling without replacement. A detailed description of the sampling process is available elsewhere^(16)^.

The short reference period used for recording expenditures on eating in each household (7 d) does not allow identifying the usual food purchase pattern in each household. Thus, the unit of analysis of the present study consisted of clusters of households belonging to each of the 550 sampling strata from the 2008–2009 HBS, ensuring units of study with a wide range of geographic and socio-economic variation, in which precise consumption information can be known. Households with family income smaller than 1 minimum wage or greater than 40 minimum wages were excluded from this database to match the NSCPI database. Therefore, the final sample was composed from 550 strata, involving 51 709 households.

Interviews were carried out throughout the year in order to replicate the seasonal variation in household expenditures and incomes in each stratum. The main information of the 2008–2009 HBS used in the present study was data on foods and beverages acquired for household consumption, registered in an electronic booklet by the head of the household supported by a trained interviewer (for this, daily visits were conducted in the households). Detailed information was registered for each acquisition, such as the name of the product, the amount acquired (in shopping units and in grams or millilitres), the total value of the expenditure and the outlet where the purchase was made (such as supermarket, hypermarket, bakery, greengrocery, convenience store). Data on a total of 1700 foods and beverages were available (except for brand information). Costs were deflated considering a reference date in the middle of the collection period (15 January 2009)^(16)^. The total acquired amounts and the costs were divided by seven and by the number of individuals in the household to express daily per capita consumption and a proportional expenditure value. Food prices (in R$/kg) were estimated by dividing the total expended in the household strata by the total quantity of each product acquired.

A list containing the mean daily per capita quantity and price of each item was then created to be used in the novel data set.

### Variation in monthly food price (price index) data

The NSCPI is responsible for the continuous and systematic estimation of consumer price indexes, having commercial (and service) facilities as data collection units. The consumer price index of interest in the present study was the Extended Consumer Price Index (E-CPI), in which the target population covers Brazilian families with monthly incomes ranging from 1 to 40 minimum wages (regardless of the source of income)^(17)^. This index has been calculated since October 1980 always through a framework of consumption provided by the most recent HBS available^(17,18)^. Price data for the E-CPI are collected from the 1st to the 30th day of each month, and the monthly index results from the comparison of the current prices with those from the 30 d of the preceding period^(17)^. More information regarding the data collection and calculation of the price index is available elsewhere^(17,18)^. Only data on the monthly variation of price are available (as a percentage of the value measured in the preceding month).

The E-CPI series from January 1995 to December 2017 was analysed. Information collected prior to July 1989 was not available on the Brazilian Institute of Geography and Statistics’ website and could not be included. The information from July 1989 to December 1994 was intentionally excluded due to the intense economic crisis experienced in the country from the late 1980s to the early 1990s, impacting on both data quality and price changes. This scenario led to the adoption of a new currency in Brazil on 27 February 1994 (the Real, R$), which is still in use at the present time (2019).

Information was used in the most disaggregated way possible. Data on 152 items, predominantly concerning a single product, were initially available. Of these, forty-six were discarded due to insufficient data (price series available was for a reduced period of time, less than 5 years, or had several months missing). Infusions (ground coffee and mate tea) and alcoholic beverages (beer and unspecified alcoholic beverages) were also excluded since their pattern of consumption differs from that of the other items on the list. Finally, 102 items (foods or beverages) with complete information for the period (1995–2017) were included in the present study.

### Linkage process, classification of foods and beverages, and price series estimation

The E-CPI product list served as the basis for the novel data set since it contained a smaller, more aggregated and frequently not well described (that could cover a range of different foods, e.g. ‘biscuits’) collection of items. A qualitative process was used to determine the most appropriate 2008–2009 HBS match for each of the 102 items from the E-CPI list. For the items aggregated in the E-CPI list, a similar aggregation was conducted for the HBS data. Where multiple HBS items were deemed a suitable match for an E-CPI item, the most purchased product was used as the price series reference (e.g. ‘cracker’). This procedure was initially conducted independently by two researchers and both lists were compared (*κ* > 0·98, excellent agreement). Disagreements were judged by a third researcher.

The NOVA food classification^(19)^ was then employed. Foods were divided into four groups and their respective subgroups: (i) unprocessed or minimally processed foods (meats; milk and eggs; vegetables; fruits; roots and tubers; cereals and pulses); (ii) processed culinary ingredients (vegetable and animal fats; sugar; salt); (iii) processed foods (processed meats; processed vegetables; French bread); and (iv) ultra-processed foods (confectionery; sausages; cakes, bread and crackers; soft drink; other ultra-processed foods). Considering that unprocessed or minimally processed foods are usually consumed with processed culinary ingredients, the price of these groups combined was calculated. Processed foods included canned or bottled fruits and vegetables; sweetened fruit pastes; salted or canned meats; canned fish; and artisanal breads. Flavoured yoghurts; mayonnaise; biscuits; margarine; ice cream; chocolate and others were considered ultra-processed foods.

Beginning with the price of each product in January 2009 (from the 2008–2009 HBS), the current price series (or ‘nominal price series’) was calculated for each product using two formulas: *A* = *B* × [1 + (*C*/100)] for the months after January 2009; and *A* = *B*/[1 + (*C*/100)] for the months before January 2009^(20)^, where *A* is the nominal price in the current month, *B* is the nominal price in the base month (or the nominal price calculated in the previous month of the sequence) and *C* is the price index in the current month. The deflated price series (or ‘real price series’) of each of these products was also calculated, using the following formula: *D* = (*E*/*F*) × *G*^(20)^, where *D* is the real price in the current month, *E* is the index number of the food category in the base month (official price inflation data)^(21)^, *F* is the index number of the food category in the current month and *G* is the nominal price in the current month. December 2017 was considered the base month for the calculation of the real price series. Mean price of each food group and subgroup was estimated based on a weighted mean of the price of its constituents (weighted by the amount acquired (in kilograms) of each item according to 2008–2009 HBS).

### Statistical analysis

Initially, the mean price of each group and subgroup and the 95 % confidence interval were estimated for the entire study period and according to three periods of time: 1995–2002, 2003–2010 and 2011–2017. The ANOVA test was used to compare the value between the periods. The monthly price of each group and its main subgroups was then plotted to analyse changes in prices for the period from 1995 to 2017. Fractional polynomial models were employed to synthesize price changes and also to forecast the price of each food group and subgroup up to 2030. Polynomials of the first to fifth degree were evaluated and the one with the highest *R*^2^ was chosen. Besides that, the relative prices among healthy foods (unprocessed or minimally processed foods and processed culinary ingredients) and unhealthy foods (ultra-processed foods) were calculated, based on real and estimated price series of both groups.

The central assumption is that the determinants of price changes observed in the period from 1995 to 2017 will continue their behaviour in the following period, supporting the reduction of the difference between the price of healthy and unhealthy foods observed in the period in which the data were measured.

The statistical software package Stata version 14.1 was used in the organization and analysis of the data.

## Results

From 1995 to 2017, processed foods were the most expensive group (R$ 7·64/kg), followed by ultra-processed foods (R$ 6·92/kg), then unprocessed or minimally processed foods and processed culinary ingredients (R$ 4·28/kg; Table 1). The food subgroups with the highest prices were processed meats (R$ 16·75/kg), confectionery (R$ 16·40/kg), sausages (R$ 11·14/kg) and meats (R$ 11·07/kg), and the lowest prices were for salt (R$ 1·22/kg), sugar (R$ 2·22/kg) and soft drink (R$ 2·81/kg; Table 1).

**Table 1:** Mean prices† (with 95 % confidence intervals) of unprocessed or minimally processed foods and processed culinary ingredients, processed and ultra-processed foods according to three time periods. Brazil‡, 1995–2017

	Price (R$/kg) according to time periods							
	1995–2002		2003–2010		2011–2017		Total	
Food group/subgroup	Mean	95 % CI	Mean	95 % CI	Mean	95 % CI	Mean	95 % CI
Unprocessed or minimally processed foods and processed culinary ingredients	3·75	3·71, 3·80	4·43	4·41, 4·46	4·70*	4·66, 4·73	4·28	4·23, 4·33
Unprocessed or minimally processed foods								
Meats	9·08	8·89, 9·27	11·28	11·10, 11·46	13·10*	13·00, 13·20	11·07	10·85, 11·28
Milk and eggs	3·89	3·82, 3·95	4·50	4·44, 4·55	4·58	4·52, 4·63	4·31	4·26, 4·36
Vegetables	3·64	3·56, 3·72	4·16	4·05, 4·27	4·89*	4·75, 5·02	4·20	4·11, 4·28
Fruits	3·13	3·06, 3·20	3·09	3·05, 3·12	3·46	3·38, 3·54	3·21	3·17, 3·25
Roots and tubers	3·15	3·04, 3·25	2·99	2·89, 3·08	3·35	3·20, 3·50	3·15	3·08, 3·22
Cereals and pulses	2·97	2·88, 3·05	3·76	3·68, 3·85	3·64	3·56, 3·72	3·45	3·38, 3·51
Processed culinary ingredients								
Vegetable and animal fats	4·39	4·27, 4·50	5·64	5·49, 5·78	4·89	4·79–4·99	4·97	4·88–5·07
Sugar	1·80	1·74, 1·87	2·45	2·35, 2·55	2·44	2·35–2·53	2·22	2·16, 2·28
Salt	0·79	0·79, 0·80	1·28	1·21, 1·35	1·64*	1·62–1·66	1·22	1·17, 1·27
Processed foods	6·63	6·51, 6·76	8·05	8·01, 8·09	8·32*	8·26–8·38	7·64	7·54, 7·74
Processed meats	13·75	13·43, 14·07	17·38	17·12, 17·63	19·47*	19·33–19·62	16·75	16·44, 17·07
Processed vegetables	10·11	10·02, 10·20	11·37	11·23, 11·52	10·96	10·89, 11·04	10·81	10·72, 10·90
French bread	5·94	5·81, 6·07	7·25	7·21, 7·30	7·50*	7·43, 7·56	6·87	6·77, 6·96
Ultra-processed foods	6·75	6·69, 6·81	7·31	7·22, 7·39	6·67	6·65, 6·70	6·92	6·87, 6·97
Confectionery	15·64	15·40, 15·88	17·71	17·48, 17·95	15·75	15·64, 15·87	16·40	16·23, 16·56
Sausages	10·30	10·23, 10·38	11·81	11·68, 11·94	11·33	11·26, 11·39	11·14	11·05, 11·23
Cakes, bread and crackers	10·55	10·41, 10·68	11·71	11·59, 11·84	10·24	10·19, 10·30	10·86	10·76, 10·96
Soft drink	2·70	2·66, 2·74	2·88	2·84, 2·91	2·85	2·83, 2·86	2·81	2·78, 2·83
Other ultra-processed foods	10·22	10·12, 10·31	10·08	9·87, 10·29	8·36	8·32, 8·39	9·60	9·48, 9·73

* Mean price values were significantly different between the three periods (*P* < 0·05).

† Real price, deflated to represent December 2017 values. For further information, see the ‘Methods’ section.

‡ Based on a novel data set created by combining the 2008–2009 Household Budget Survey data and information from the National System of Consumer Price Indexes. For further information, see the ‘Methods’ section.

**Fig. 1:**
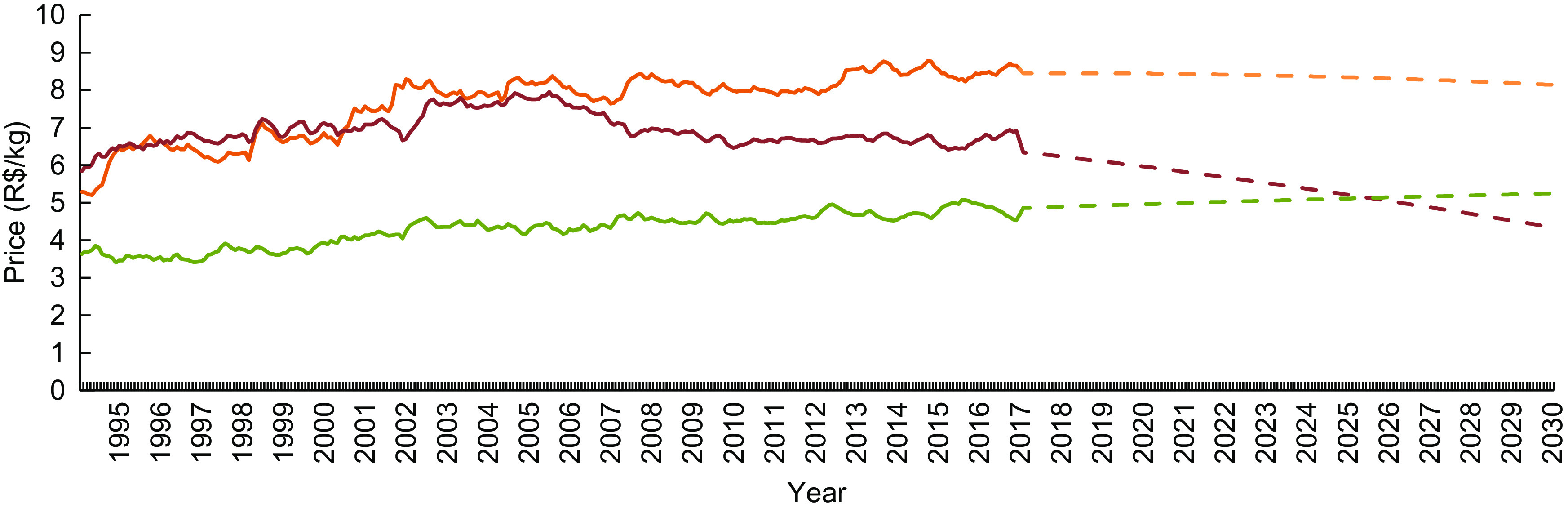
Mean monthly price† (R$/kg) of unprocessed or minimally processed foods and processed culinary ingredients (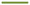), processed foods (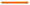) and ultra-processed foods (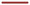) for the period from 1995 to 2017 and forecast up to 2030‡. Brazil§, 1995–2030. Observations: the dashed segment of each group represents projected price estimates. *R*^2^: 0·89 (unprocessed or minimally processed foods and processed culinary ingredients), 0·87 (processed foods), 0·55 (ultra-processed foods). †Real price from January 1995 to December 2017, deflated to represent December 2017 values. ‡From 2017 to 2030, estimated through fractional polynomial models. §Based on a novel data set created by combining 2008–2009 Household Budget Survey data and information from the National System of Consumer Price Indexes. For further information, see the ‘Methods’ section

**Fig. 2:**
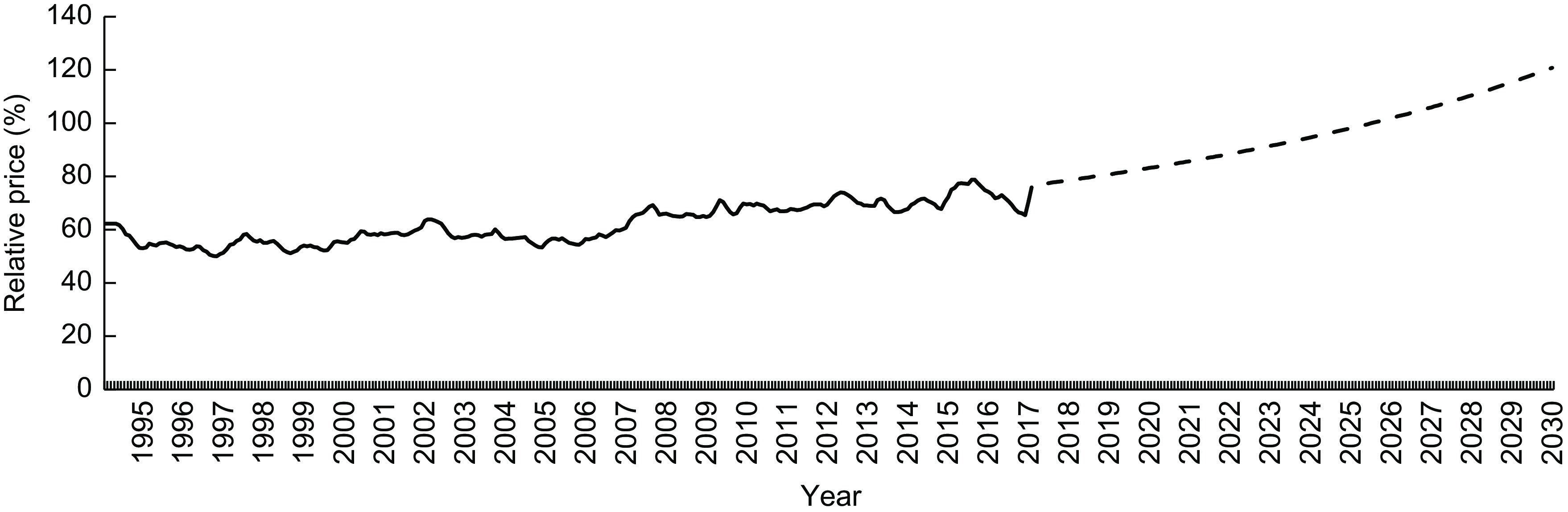
Price of unprocessed or minimally processed foods and processed culinary ingredients relative to the price of ultra-processed foods† (%) for the period from 1995 to 2017 and forecast up to 2030‡. Brazil§, 1995–2030. Observations: the dashed segment represents projected relative price estimates. †Relative prices from January 1995 to December 2017, calculated through real price of unprocessed or minimally processed foods and processed culinary ingredients *v*. ultra-processed foods. ‡Relative prices from 2017 to 2030, calculated through estimated price of unprocessed or minimally processed foods and processed culinary ingredients *v*. ultra-processed foods. §Based on a novel data set created by combining 2008–2009 Household Budget Survey data and information from the National System of Consumer Price Indexes. For further information, see the ‘Methods’ section

The price of unprocessed or minimally processed foods and processed culinary ingredients increased continuously during the period studied (from R$ 3·45/kg in 1995 to R$ 4·69/kg in 2017). The price of processed foods also increased (from R$ 5·28/kg in 1995 to R$ 8·55/kg in 2017); however, the magnitude of the increase was gradually reduced over the years. On the other hand, the price of ultra-processed foods increased during the first third of the study period (with a similar magnitude to that of processed foods) and assumed an opposite trend after the early 2000s (Fig. 1). Sustaining this trend, the forecast for 2030 indicates a further increase in unprocessed or minimally processed foods and processed culinary ingredients prices (R$ 4·69/kg in 2017 to R$ 5·24/kg in 2030), a decrease in ultra-processed foods prices (R$ 6·62/kg in 2017 to R$ 4·34/kg in 2030) and stability in processed foods prices. According to these predictions, healthy foods (unprocessed or minimally processed foods and processed culinary ingredients) will become more expensive than unhealthy foods (ultra-processed) in Brazil from 2026 (Fig. 1). The relative price of these healthy foods in relation to unhealthy foods increased over the period, from 53·08 % (1995) to 70·80 % (2017) to 120·75 % (2030; Fig. 2).

**Fig. 3:**
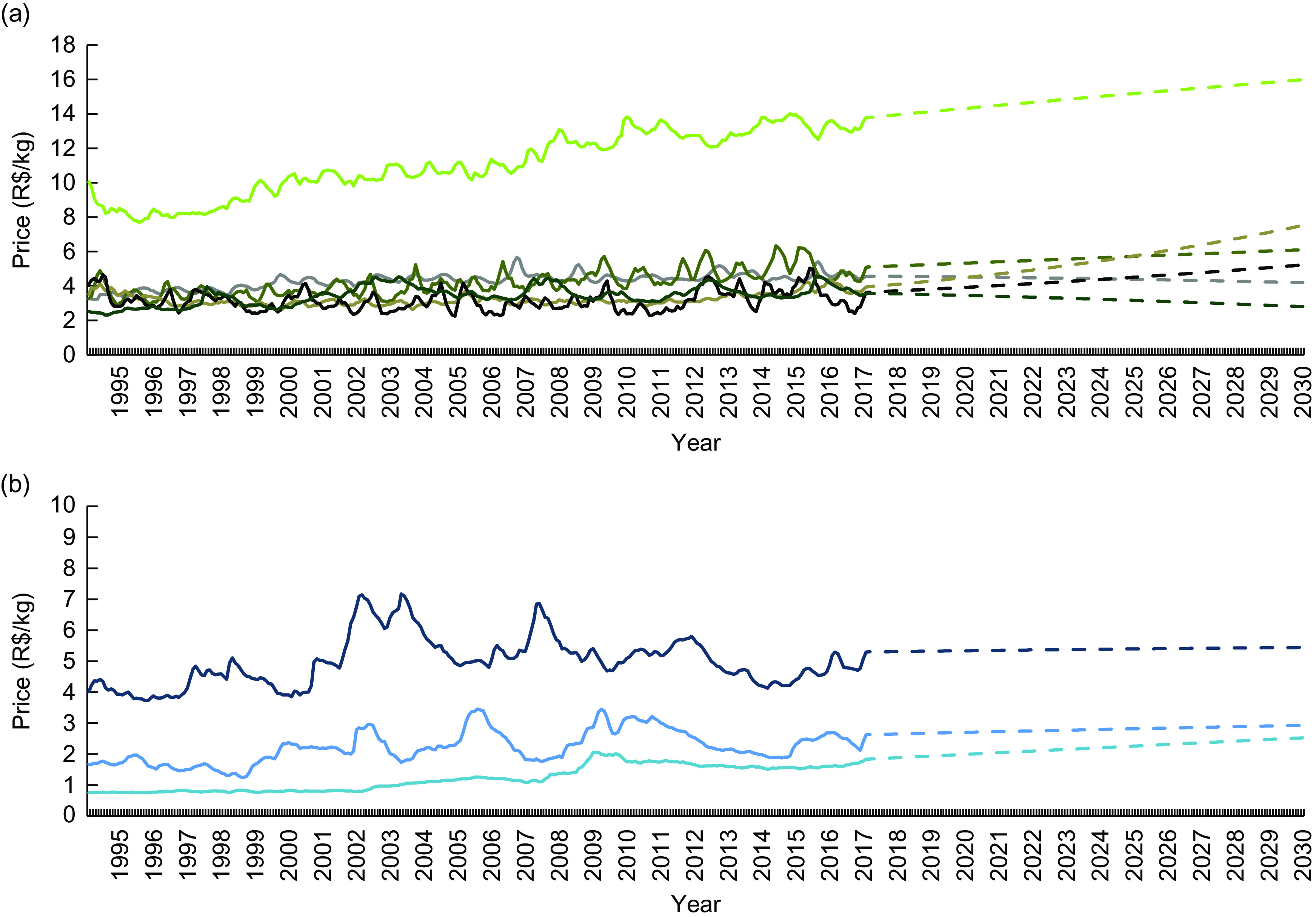
Mean monthly price† (R$/kg) of (a) unprocessed or minimally processed foods (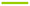, meats; 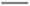, milk and eggs; 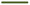, vegetables; 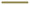, fruits; 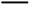, roots and tubers; 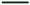, cereals and pulses) and (b) processed culinary ingredients (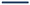, vegetable and animal fats; 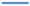, sugar; 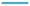, salt) for the period from 1995 to 2017 and forecast up to 2030‡. Brazil§, 1995–2030. Observations: the dashed segment of each group represents projected price estimates. *R*^2^: 0·93 (meats), 0·68 (milk and eggs), 0·52 (vegetables), 0·65 (fruits), 0·16 (roots and tubers), 0·45 (cereals and pulses), 0·15 (vegetable and animal fats), 0·30 (sugar), 0·80 (salt). †Real price from January 1995 to December 2017, deflated to represent December 2017 values. ‡From 2017 to 2030, estimated through fractional polynomial models. §Based on a novel data set created by combining 2008–2009 Household Budget Survey data and information from the National System of Consumer Price Indexes. For further information, see the ‘Methods’ section

**Fig. 4:**
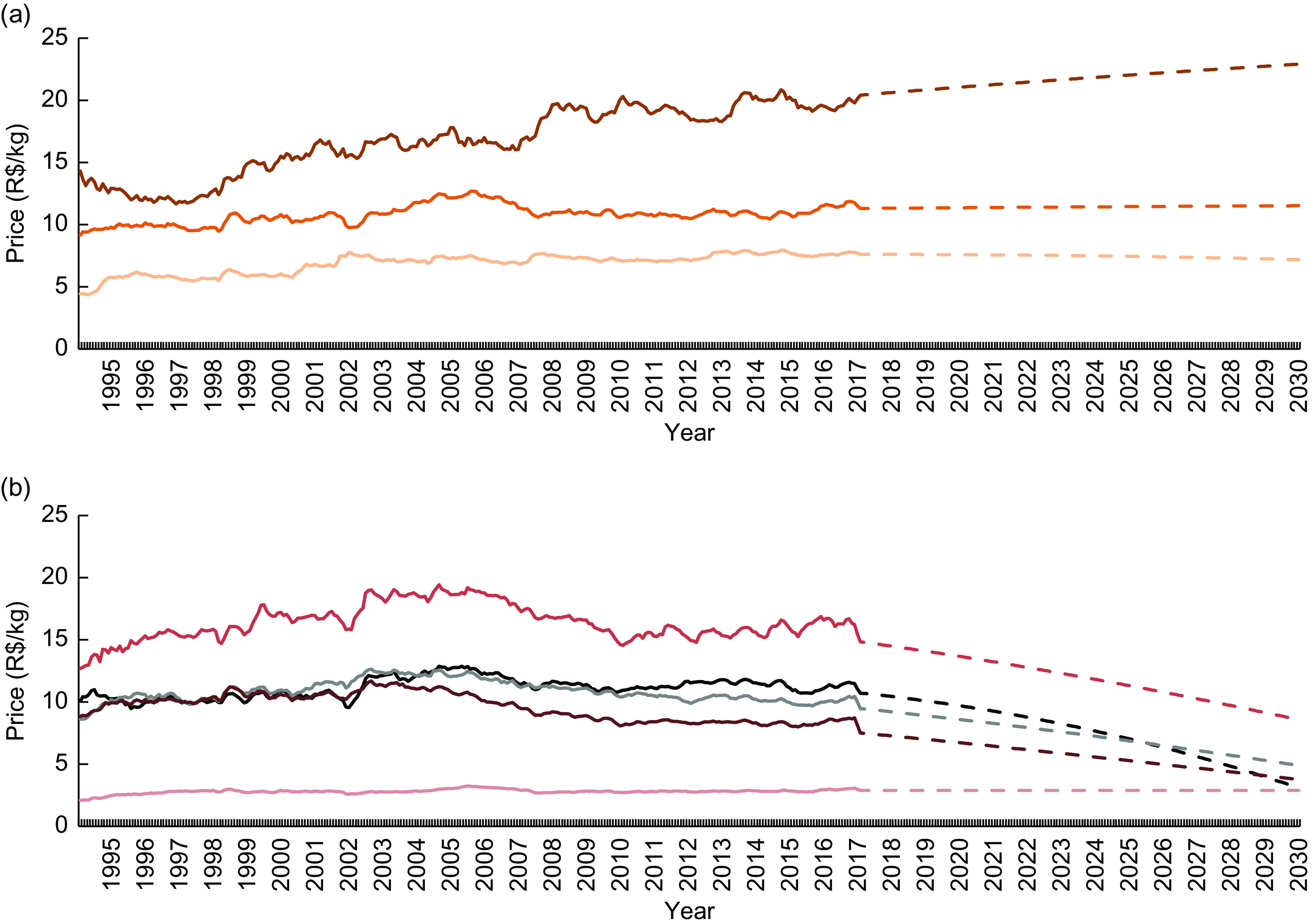
Mean monthly price† (R$/kg) of (a) processed foods (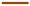, processed meat; 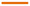, processed vegetables; 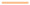, French bread) and (b) ultra-processed foods (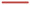, confectionery; 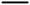, sausages; 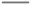, cakes, bread and crackers; 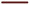, other ultra-processed foods; 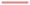, soft drink) for the period from 1995 to 2017 and forecast for 2030‡. Brazil§, 1995–2030. Observations: the dashed segment of each group represents projected price estimates. *R*^2^: 0·91 (processed meats), 0·40 (processed vegetables), 0·83 (French bread), 0·56 (confectionery), 0·48 (sausages), 0·70 (cakes, bread and crackers), 0·57 (soft drink), 0·75 (other ultra-processed foods). †Real price from January 1995 to December 2017, deflated to represent December 2017 values. ‡From 2017 to 2030, estimated through fractional polynomial models. §Based on a novel data set created by combining 2008–2009 Household Budget Survey data and information from the National System of Consumer Price Indexes. For further information, see the ‘Methods’ section

Among the unprocessed or minimally processed foods subgroups, meats had the highest mean price (R$ 11·07/kg), while roots and tubers presented the lowest price (R$ 3·15/kg; Table 1). Except for cereals and pulses and milk and eggs, all subgroups presented an ascending trend up to 2030, especially fruits (R$ 3·80/kg in 2017 to R$ 7·51/kg in 2030; Fig. 3(a)). The price of the processed culinary ingredients subgroups varied intensely during the study period; however, presented similar values in 1995 and 2017 (Fig. 3(b)). Among the processed foods, only the price of processed meats increased (R$ 12·92/kg in 1995 to R$ 20·13/kg in 2017), further influencing the difference in price between these products and others in the group (Fig. 4(a)). Finally, with the exception of soft drink, the price of all ultra-processed foods subgroups decreased between 1995 and 2017, indicating further reduction until 2030 (Fig. 4(b)).

## Discussion

The organization of data from two different sources into a novel data set allowed a comprehensive analysis of food prices in Brazil, considering the healthy eating concept adopted by the Brazilian Dietary Guidelines. Monthly data on the price of 102 foods and beverages were analysed for a period of more than 20 years (from 1995 to 2017) and forecast up to 2030. The mean prices of processed and ultra-processed foods were higher than those of unprocessed or minimally processed foods and processed culinary ingredients in the period from 1995 to 2017. The price of unprocessed or minimally processed foods and processed culinary ingredients increased continuously until 2017, while the price of processed foods also increased, however with smaller magnitude. Meanwhile, the price of ultra-processed foods increased during the first third of the study period (up to the early 2000s) and decreased thereafter. If these forecasts are met, from 2026 healthy foods (unprocessed or minimally processed foods and processed culinary ingredients) will become more expensive than unhealthy foods (ultra-processed foods) in Brazil, increasing the price gap between these groups but now unfavourable to the Brazilian Dietary Guidelines’ recommendations.

The relationship between food prices and the adoption of healthy diets has been intensely studied worldwide^(22)^. Healthier diets with high nutritional value, based on unprocessed or minimally processed foods, tend to cost more per unit of energy than less healthy diets, generally based on ultra-processed foods, both in developed^(10,11)^ and developing countries^(23,24)^.

Brazil is certainly no exception. According to a study based on data collected in the 2008–2009 HBS, unprocessed or minimally processed foods, such as fresh meat, milk, fruits and vegetables, tend to cost more per unit of energy than ultra-processed foods. However, the same study highlights that due to the low cost of grains, such as rice and beans, per unit of energy, traditional healthy diets are still cheaper than those based on ultra-processed foods^(12)^. The results of the present study indicate that these conclusions may become outdated in the near future. Although comparisons involving food prices per energy unit and per kilogram (as conducted by the present study) should be conducted carefully, the magnitude of the relative change in price identified in the present results may safely be applied to the prices per unit of energy identified in Claro *et al*.’s study^(12)^. Their data indicate a price of R$ 1·56/1000 kcal (4184 kJ) for unprocessed and minimally processed foods and processed culinary ingredients, in comparison to R$ 2·26/1000 kcal (4184 kJ) for ultra-processed foods. Based on the results of the present study, the price of unprocessed and minimally processed foods and processed culinary ingredients increased by 5·53 % (from R$ 4·50/kg to R$ 4·75/kg) between 2009 and 2017, while the price of ultra-processed foods decreased by 1·55 % (from R$ 6·90/kg to R$ 6·79/kg). When these rates of variation are applied to the price values mentioned above, it is possible to identify that the price per unit of energy of unprocessed and minimally processed foods and processed culinary ingredients is approaching the price per unit of energy of ultra-processed foods in the present day (2019). While the price per unit of energy has the advantage of being nutritionally contextualized, it has the disadvantage of not being clearly perceived by consumers. Thus, the present data complement this information by predicting that, in less than a decade (2026), the economic disadvantage in the consumption of healthy diets based on unprocessed and minimally processed foods and processed culinary ingredients will be visible in the shelf prices.

Although the present study is the first one to analyse price trends in the context of the Brazilian Dietary Guidelines’ recommendations, evidence suggesting that healthy diets were becoming more expensive in the country was already available. A study based on price data for the municipality of São Paulo, the most populated city of the country with more than 12 million inhabitants, indicated an increase in the price of fruits and vegetables and a decrease in the price of fats, oils, condiments, sugars and processed foods (industrialized foods) between 1939 and 2010^(13)^.

The present study does not reveal the exact cause of the observed changes, but some factors can be listed as potentially responsible. First, Brazil’s last burst of economic growth coincided with the change in the food price scenario seen in the results. This economic growth fomented technological improvements in industry, resulting in greater productivity and lower production costs over time^(25)^. Although some of these improvements also apply to the production of unprocessed or minimally processed food items, they are certainly more effective in the Big Food and Big Soda industries, where successive rounds of food processing benefit from it^(6,7)^. Second, the production of unprocessed or minimally processed food relies less on governmental incentives in Brazil than the production of ultra-processed foods. National agricultural policy is still organized in a way that favours the production of commodities such as corn, soya and sugarcane, as these items and their by-products, such as soya oil, animal feed, sugar and ethanol, have a central role in the commercial and economic life of the country^(26)^. Since these items are widely used in the production of ultra-processed foods the food industry benefits from this scenario^(6)^. It should also be mentioned that policies directed towards family farmers, responsible for the production of about 70 % of all foods consumed in Brazilian households (according to the data of the last agricultural census^(27)^), have also undergone an intensification since the early 2000s. However, these actions have lost strength in recent years, jeopardizing more than a decade of incentives for the development of a new agricultural scenario in the country. Third, still in relation to the governmental environment, a notorious amount of questionable fiscal benefits have historically been granted to the Big Soda and Big Food industries in Brazil, a practice that also intensified in the past decade^(28,29)^.

The increase in the consumption of ultra-processed foods and the reduction in the consumption of unprocessed or minimally processed foods is already a reality in Brazil^(30)^. Between 1995 and 2009 (the period included in our study for which household food acquisition data were available), the consumption of ultra-processed foods increased from 21·0 to 29·6 % of total energy, while the consumption of unprocessed or minimally processed foods decreased from 44·2 to 38·9 %^(8)^. Ultra-processed products are dominating the global food system due to their profitability for the large multinational companies^(6)^. Thus, these companies provide promotions and volume discounts to induce retailers to shift to their products and win consumers with lower product prices^(7)^. In addition to the aggressive marketing, the convenience and the hyper-palatability of these ultra-processed foods^(31)^, the price scenario identified (and predicted) in the present study will certainly intensify this consumption trend, and consequently increase the consumption of unhealthy diets and the risk for NCD. In 2017, NCD were already responsible for approximately three-quarters (73 %) of all deaths and more than half (62 %) of the total years of healthy life lost due to premature death and disability worldwide^(32)^.

Changes in food prices are an important step towards improving population health and actions in this direction have been long recommended by international organizations such as the WHO^(33)^. Policies aimed at food prices should encourage the consumption of healthy, unprocessed or minimally processed foods and discourage the consumption of unhealthy ultra-processed foods. The potential impact of strategies to change the food price scenario in relation to deaths from cardiometabolic diseases was recently modelled for the US population^(4)^. Using nationally representative data, a comparative risk assessment was performed to model the potential effects on cardiometabolic disease deaths and disparities of price subsidies (10 %, 30 %) for fruits, vegetables, whole grains and nuts/seeds and taxes (10 %, 30 %) on processed meat, unprocessed red meats and sugar-sweetened beverages. All the interventions would reduce deaths from cardiometabolic diseases, with large reductions in stroke, followed by diabetes and CHD. Jointly altering the prices of all seven dietary factors (10 % each) would prevent 23 174 deaths from cardiometabolic diseases per year and an even greater impact would be achieved in the case of a 30 % change^(4)^.

Such intense change is hardly achievable in the short term, especially in relation to the reduction of the prices of healthy foods, which generally involves the need for large financial investments by the government. In Brazil, federal taxes were removed on the products of the basic food basket in 2013, including some fresh foods^(34)^. Nevertheless, this measure had little impact on the population’s food consumption or even on the price scenario^(35)^. Thus, the debate in countries has been more inclined towards making unhealthy foods less affordable through increasing taxes, having a mandatory minimum unit price or restricting price discounts for ultra-processed foods^(24,36)^. Taxation has been the strategy most employed^(37)^, possibly due to its application and enforcement being simpler than the other options. Current evidence supports the implementation of taxes that increase the price of products by 20 % or more to reduce the consumption of unhealthy foods^(36)^. The WHO already indicated this measure for sweetened beverages in 2016^(38)^ and several countries have considered or already adopted this measure, such as Mexico (with 10 % tax on sweetened beverages and 8 % tax on ultra-processed foods with energy density above 1151 kJ (275 kcal)/100 g)^(23)^ and Chile (in 2014, the tax for beverages with an added sugar concentration above 6·25 g/100 ml increased from 13 to 18 %)^(39)^. In Brazil, several bills on the subject are under discussion in the National Congress; however, due to strong resistance from the Big Soda industry and some groups of Federal Legislators (largely financed by the Big Food and Big Soda industries), little progress has been made so far^(40)^. Although our results have shown a small increase in the price of soft drinks, they remain the cheapest ultra-processed product on the market, suggesting that the adoption of taxation could be an effective way to discourage its consumption in the country.

Some limitations of the present study should be considered. Different approaches to measure food prices can produce conflicting results regarding the cost of healthy diets^(41)^. Prices per unit of energy tend to be highly influenced by the energy density of foods, sometimes resulting in data that are difficult to interpret (such as for low-calorie foods and beverages). Therefore, the real price series were calculated considering price per unit of weight (R$/kg), in order to decrease this bias and to provide information beyond the nutritional perspective. However, analyses involving food prices per energy unit (R$/1000 kcal; 1 kcal = 4·184 kJ) are presented in the online supplementary material. Another limitation to consider is that the number of foods and beverages included in the study was small (*n* 102) and only reflects those foods included in the E-CPI, rather than a full range of items available on the Brazilian market. However, the items included in the analysis reflect those items most commonly purchased by the Brazilian population (approximately 63 % of the total energy acquired), considering the role of the E-CPI as a measure of consumer inflation. The present study also did not account for variations in price by outlet type (since no specific information was available) and assumed the quantity of each item acquired to be constant in the studied period (based on the 2008–2009 HBS, since changes in the structure of national HBS between 2002–2003 and 2008–2009 editions restrict comparability and more recent information is unavailable for the Brazilian population). A final limitation concerns product aggregation in the E-CPI database, which made it impossible to produce individualized estimates for each of the products. The Brazilian Institute of Geography and Statistics states that only products with similar inflation behaviour are aggregated^(17,18)^; however, this is not verifiable, based on the data available.

## Conclusion

In conclusion, the results illustrated significant changes in the food and beverage price scenario in Brazil between 1995 and 2017, which shall extend until at least 2030. This certainly imposes an important barrier for adoption of the Brazilian Dietary Guidelines. Monitoring food prices over time complements the country’s health monitoring framework and helps to improve nutritional strategies and fiscal policies, in order to encourage a healthier diet and the prevention of NCD in the population. Further research should be conducted to identify the ideal combination of interventions to maximize ultra-processed food prices and to reduce fresh food prices.

## Supplementary material

For supplementary material accompanying this paper visit https://doi.org/10.1017/S1368980019003586. click here to view supplementary material
