# Supplementary material for: What to expect from the price of healthy and unhealthy foods over time? The case from Brazil
Source: Public Health Nutr. 2020 Jan 15;23(4):579–88. doi: 10.1017/S1368980019003586 (PMC7058424; doi:10.1017/S1368980019003586)
Supplement: Supplementary file 1 [file S1368980019003586sup001.docx]

**SUPPLEMENTARY MATERIAL**

Analyses involving per calorie food prices were available in this online supplementary material. The real price series per calorie (R$/1,000Kcal) were estimated in a similar way to the real price series per kilograms (R$/Kg). However, the total daily amount of each food was converted into energy (Kcal), using the TACO table (Brazilian Food Composition Table)^(1)^ or the official table of nutritional composition of the United States^(2)^. The amount of these foods and beverages in calories was used to weigh the mean monthly price (R$/1,000kcal) for each food group and subgroup.

References

1. UNICAMP (2011) Universidade Estadual de Campinas. Núcleo de Estudos e Pesquisas em Alimentação (NEPA). Tabela Brasileira de Composição de Alimentos (TACO). UNICAMP, Campinas. <http://www.nepa.unicamp.br/taco/contar/taco_4_edicao_ampliada_e_revisada.pdf?arquivo=taco_4_versao_ampliada_e_revisada.pdf> (accessed February 2019).
2. USDA (2010) United States Department of Agriculture. USDA National Nutrient Database for Standard Reference, 23 ed. U.S. Department of Agriculture, USDA, Washington. <https://ndb.nal.usda.gov/ndb/search/list> (accessed February 2019).

**Figure S1.** Mean monthly price^a^ (R$/1,000Kcal) and of unprocessed or minimally processed foods and processed culinary ingredients, processed foods and ultra-processed foods for the period from 1995 to 2017 and forecast for 2030^b^. Brazil^c^, 1995-2030.

^a^Real price from January 1995 to December 2017, deflated to represent December 2017 values. For further information, see the Methods section.

^b^From 2017 to 2030, estimated through fractional polynomial models. For further information, see the Methods section.

^c^Based on a novel dataset created by combining 2008-09 HBS data and information from the NSCPI. For further information, see the Methods section.

Observations: The dashed segment of each group represents projected price estimates. R²: 0.88 (unprocessed or minimally processed foods and processed culinary ingredients), 0.87 (processed foods), 0.55 (ultra-processed foods).

**Figure S2.** Price of unprocessed or minimally processed foods and processed culinary ingredients relative to the price of ultra-processed foods (%)^a^ for the period from 1995 to 2017 and forecast for 2030^b^. Brazil^c^, 1995-2030.

^a^Relative prices from January 1995 to December 2017, calculated through of real price of unprocessed or minimally processed foods and processed culinary ingredients versus ultra-processed foods. For further information, see the Methods section.

^b^Relative prices from 2017 to 2030, calculated through of estimated price of unprocessed or minimally processed foods and processed culinary ingredients versus ultra-processed foods. For further information, see the Methods section.

^c^Based on a novel dataset created by combining 2008-09 HBS data and information from the NSCPI. For further information, see the Methods section.
